# Supplementary material for: Factors Affecting Shark Detection from Drone Patrols in Southeast Queensland, Eastern Australia
Source: Biology (Basel). 2022 Oct 23;11(11):1552. doi: 10.3390/biology11111552 (PMC9687398; doi:10.3390/biology11111552)
Supplement: Supplementary file 1 [file biology-11-01552-s001.zip › biology-1915311-supplementary.pdf]

Supplementary material

Table S1. Akaike Information Criterion (AIC) values for the Generalised Linear Mixed Models run to quantify the effect of operational and environmental factors on the probability of sighting sharks.

Model	AIC value
Shark presence/absence ~ Location + flight no. + wind speed + wind direction + cloud cover + atmospheric pressure + sea state + turbidity + glare + season + presence of other fauna + 7 day rainfall	616.55
Shark presence/absence ~ Location + flight no. + wind speed + wind direction + cloud cover + atmospheric pressure + sea state + turbidity + glare + season + presence of other fauna	616.71
Shark presence/absence ~ Location + flight no. + wind speed + wind direction + cloud cover + atmospheric pressure + sea state + turbidity + glare + season	622.04
Shark presence/absence ~ Location + flight no. + wind speed + wind direction + cloud cover + atmospheric pressure + sea state + turbidity + glare	632.72
Shark presence/absence ~ Location + flight no. + wind speed + wind direction + cloud cover + atmospheric pressure + sea state + turbidity	624.94
Shark presence/absence ~ Location + flight no. + wind speed + wind direction + cloud cover + atmospheric pressure + sea state	628.46
Shark presence/absence ~ Location + flight no. + wind speed + wind direction + cloud cover + atmospheric pressure	621.94
Shark presence/absence ~ Location + flight no. + wind speed + wind direction + cloud cover	620.76
Shark presence/absence ~ Location + flight no. + wind speed + wind direction	632.08
Shark presence/absence ~ Location + flight no. + wind speed	622.07
Shark presence/absence ~ Location + flight no.	622.99
Shark presence/absence ~ Location	636.19
***Shark presence/absence ~ Location + flight no. + season + presence of other fauna**	**612.19**

***best model which includes only significant variables and has the lowest AIC.**
